# Rapid effects of deep brain stimulation reactivation on symptoms and neuroendocrine parameters in obsessive-compulsive disorder

**DOI:** 10.1038/tp.2015.222

**Published:** 2016-01-26

**Authors:** P P de Koning, M Figee, E Endert, P van den Munckhof, P R Schuurman, J G Storosum, D Denys, E Fliers

**Affiliations:** 1 Department of Psychiatry, Academic Medical Center, University of Amsterdam, Amsterdam, The Netherlands; 2 Department of Endocrinology and Metabolism, Academic Medical Center, University of Amsterdam, Amsterdam, The Netherlands; 3 Department of Neurosurgery, Academic Medical Center, University of Amsterdam, Amsterdam, The Netherlands; 4 The Netherlands Institute for Neuroscience, Royal Netherlands Academy of Arts and Sciences, Amsterdam, The Netherlands

## Abstract

Improvement of obsessions and compulsions by deep brain stimulation (DBS) for obsessive-compulsive disorder (OCD) is often preceded by a rapid and transient mood elevation (hypomania). In a previous study we showed that improvement of mood by DBS for OCD is associated with a decreased activity of the hypothalamus–pituitary adrenal axis. The aim of our present study was to evaluate the time course of rapid clinical changes following DBS reactivation in more detail and to assess their association with additional neuroendocrine parameters. We included therapy-refractory OCD patients treated with DBS (>1 year) and performed a baseline assessment of symptoms, as well as plasma concentrations of thyroid-stimulating hormone (TSH), prolactin, growth hormone, copeptin and homovanillic acid. This was repeated after a 1-week DBS OFF condition. Next, we assessed the rapid effects of DBS reactivation by measuring psychiatric symptom changes using visual analog scales as well as repeated neuroendocrine measures after 30 min, 2 h and 6 h. OCD, anxiety and depressive symptoms markedly increased during the 1-week OFF condition and decreased again to a similar extent already 2 h after DBS reactivation. We found lower plasma prolactin (41% decrease, *P*=0.003) and TSH (39% decrease, *P*=0.003) levels during DBS OFF, which increased significantly already 30 min after DBS reactivation. The rapid and simultaneous increase in TSH and prolactin is likely to result from stimulation of hypothalamic thyrotropin-releasing hormone (TRH), which may underlie the commonly observed transient mood elevation following DBS.

## Introduction

Deep brain stimulation (DBS) is an effective treatment for therapy-resistant obsessive-compulsive disorder (OCD), with a mean 50% improvement of obsessive-compulsive symptoms.^1^ After finding optimal DBS parameter settings, improvement in obsessions and compulsions usually occurs within days to weeks.^2^ This clinical response is often preceded by a rapid (minutes to hours) transient mood elevation or even hypomania (Luigjes J *et al.* Hypomania as side effect of DBS in psychiatric patients, submitted). These rapid mood alterations have been suggested to predict a long-term response of DBS for OCD. For example, Haq *et al.*^3^ reported intraoperative stimulation-induced smile and laughter induction in DBS of the anterior limb of the internal capsule and the nucleus accumbens region to predict long-term OCD response. To date, no study has systematically investigated the rapid effects of DBS on psychiatric symptoms. Moreover, the underlying mechanism of this transient mood elevation remains unknown.

Recently, we showed that mood improvement following long-term DBS of the accumbal area is strongly correlated with decreased hypothalamic–pituitary–adrenal axis activity, reflected by decreased urinary free cortisol levels.^4^ Although the causality of these findings remains elusive at this stage, this mood improvement may well result from inhibition of hypothalamic corticotropin-releasing hormone. Thus, the hypothalamus may also be involved in the mediation of acute clinical effects of DBS.

The aim of this study was to evaluate rapid clinical changes (OCD, anxiety and mood) following DBS and to assess various other neuroendocrine parameters related to the hypothalamic–pituitary axis as well as their association with DBS-induced rapid clinical changes.

## Materials and methods

### Study participants

We included 16 treatment-refractory OCD patients treated with DBS targeted at the border between the accumbens core and the ventral part of the internal capsule (Denys *et al.*^2^). Participants were chosen from a larger clinical sample of DBS-treated OCD patients and were included if they had been treated with DBS for more than 1 year. Inclusion and exclusion criteria are described in detail in the study by de Koning *et al.*^4^ Participants provided written informed consent before participation, and the local ethics committee approved this study. We included seven women and nine men aged between 32 and 56 years. The mean duration of illness was 25.9 years (range 8-48 years). Six participants had one or more comorbid disorders: major depressive disorder (*N*=4), dysthymia (*N*=1) and panic disorder (*N*=1). Nine participants had been medication free for more than 1 year. Seven participants used psychotropic medication (three clomipramine, three selective serotonin reuptake inhibitors and one atypical antipsychotic). Five participants smoked cigarettes. None of the participants suffered from metabolic, endocrine or inflammatory disorders. DBS parameters varied between participants in voltage (3.5–6.2 volts), frequency (90–150 Hz) and pulse width (130–185 μs). Medication was continued during the study. Participants were not allowed to consume alcohol, coffee and nicotine or perform excessive exercise 24 h before each measure.

One participant dropped out during the study phase and was therefore excluded. As a result, we used data from 15 out of 16 participants for the final analysis.

### Procedure

**Figure 1:**
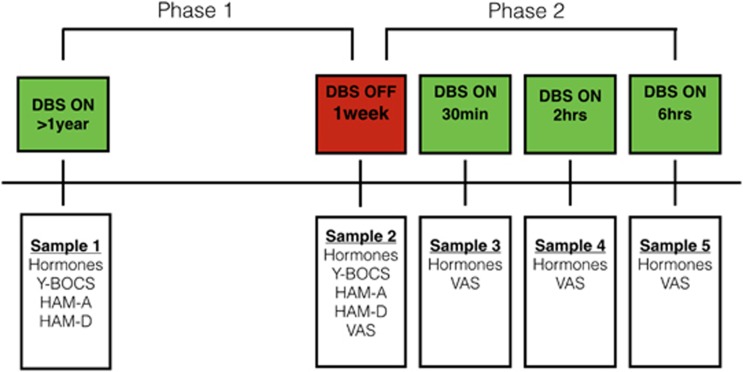
Study design. Hormones: thyroid-stimulating hormone, prolactin, growth hormone and copeptin. HVA, homovanillic acid; VAS, visual analog scale.

The study had a two-phase design (see Figure 1).

In phase 1 we measured psychiatric symptoms and neuroendocrine parameters when the DBS had been constantly activated for at least 1 year (DBS ON) and then after 1 week of DBS discontinuation (DBS OFF). Three blood samples (sample 1, DBS ON) were collected in 4.5-ml tubes (one ice-chilled EDTA, one plain and one heparin tube). These samples were collected in the morning (1000 hours) from a peripheral intravenous catheter. The peripheral intravenous catheter had been placed more than 1 h in advance of drawing the blood sample, and all participants had rested for a minimum of 1 h to minimize the effects of stress and physical activity. The following day, the DBS was turned off. After 1 week of DBS cessation, the second blood sample (sample 2, DBS OFF) was taken in the morning, following the same peripheral intravenous catheter procedure. After each blood sample collection, the samples were centrifuged for 10 min at 3000 r.p.m. Next, the plasma was pipetted and stored in 0.5-ml aliquots at −50 °C.

Following each sample collection, a trained psychiatrist assessed obsessive-compulsive, anxiety- and depressive symptoms with the Yale–Brown Obsessive-Compulsive Symptom Scale (Y-BOCS), the Hamilton Anxiety Rating Scale (HAM-A) and the Hamilton Depression Rating Scale (HAM-D).^5, 6, 7^

In phase 2, we investigated the acute effects of DBS reactivation. After 1 week of DBS discontinuation and the collection of sample 2, the DBS was reactivated. We then took three consecutive samples after 30 min (sample 3), 2 h (sample 4) and 6 h (sample 5). As the HAM-A, HAM-D and Y-BOCS are not applicable for measuring acute symptom changes, we used visual analog scale (VAS) scores in this phase to measure clinical effects. During each of these four blood samples every participant assessed his/her actual level of anxiety, depression, obsessive thoughts and need to perform compulsions through a 10-point VAS scoring (0=no symptoms–10=most symptoms ever experienced).

### Data acquisition and analysis

We investigated the long-term as well as the acute effects of DBS on plasma thyroid-stimulating hormone (TSH), prolactin, growth hormone (GH), copeptin (a stable peptide derived from the vasopressin precursor reflecting vasopressin plasma concentrations^8^) and the dopamine metabolite homovanillic acid (HVA). TSH, prolactin and GH were determined with a solid-phase time-resolved fluoroimmunoassay (Delfia, PerkinElmer, Wallac Oy, Turku, Finland). Detection limits were as follows: TSH 0.01 mU l^−1^, prolactin 0.1 μg l^−1^, GH 0.1 mU l^−1^. Copeptin was measured using an immunoluminometric assay (BRAHMS CT-proAVP LIA, BRAHMS, Thermo Scientific, Henningsdorf, Germany); detection limit was 0.4 pmoll^−1^. All assays were performed in heparin plasma with a total assay variation of <10%. Plasma levels of HVA were determined using liquid chromatography (Shimadzu, Eindhoven, the Netherlands) and electrochemical detection (DECADE 1 equipped with a VT03 cell at a potential setting of 700 mV versus Ag/Agcl reference electrode at 40 °C; ANTEC Leyden, Zoeterwoude, the Netherlands). The sensitivity of the method for HVA was 2 ng ml^−1^ plasma, and the coefficient of variations was less then 6%.

### Statistical analysis

Normality of the data was assessed using the Kolgomorov–Smirnov test. In phase 1, if normality was violated, Wilcoxon signed-rank tests were performed in place of Student's *t*-tests. For phase 2, we performed a repeated measures analysis of variance. Data are expressed as mean±s.d. or median (min–max), and results are considered significant at *P*<0.05 (two-tailed). Correlation analyses were performed applying Pearson's correlation. All statistical tests were computed with SPSS for Windows 21.0 (SPSS, Chicago, IL, USA).

## Results

### Psychiatric symptoms

Ten out of fifteen participants were initial DBS responders defined as a minimum 35% Y-BOCS decrease (mean ΔY-BOCS=17.0±6.0) after >1 year of DBS compared with pre-DBS Y-BOCS scores (mean PRE DBS Y-BOCS=33.1±3.4). The other five participants were non-responders defined as showing <25% decrease on the Y-BOCS (mean ΔY-BOCS=6.2 ±5.2 s.d.).

#### DBS ON→DBS OFF

DBS cessation for 1 week compared with DBS ON was related to an increase from mild–moderate to severe obsessive-compulsive symptoms (Y-BOCS=19.7±7.0 versus 28.6±5.1, *P*=0.001). The mean anxiety symptoms increased from mild to severe after 1-week DBS cessation (HAM-A=16.3±9.1 versus 30.2±10.2, *P*=0.000). The mean depressive symptoms increase from mild to severe (HAM-D=13.8±8.7 versus 26.0±8.9, *P*=0.002).

#### *DBS* reactivation

**Figure 2:**
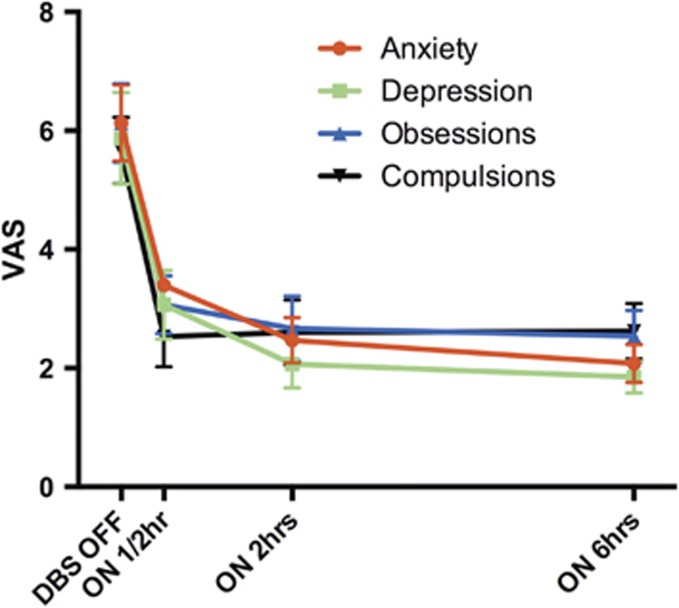
Acute effects of deep brain stimulation (DBS) reactivation (phase 2) on anxiety, depression, obsessions and compulsions using the mean visual analog scale (VAS) scores (±s.e.).

Thirty minutes after DBS reactivation we observed a mean of 51% (*P*=0.002) reduction on obsessions and 55% (*P*=0.001) reduction on need to perform compulsions (VAS scores; Figure 2). Six hours after reactivation there was a further decrease in obsessions to a total of 59% (*P*=0.003), whereas compulsions did not decrease any further but remained 54% lower than DBS OFF (*P*=0.005). DBS reactivation resulted in a 44% (0.002) reduction on anxiety symptoms (VAS score) after 30 min, mounting to a 64% reduction (*P*=0.002) after 6 h. DBS reactivation resulted in a 48% reduction (*P*=0.005) on depressive symptoms (VAS score) after 30 min, mounting to 69% (*P*=0.005) after 6 h.

Eight out of ten of the initial DBS responders and three out of five non-responders reported >50% improvement of obsessive-compulsive, anxiety and depression VAS scores within 2 h after DBS reactivation.

### Neuroendocrine changes

#### DBS ON → DBS OFF

**Figure 3:**
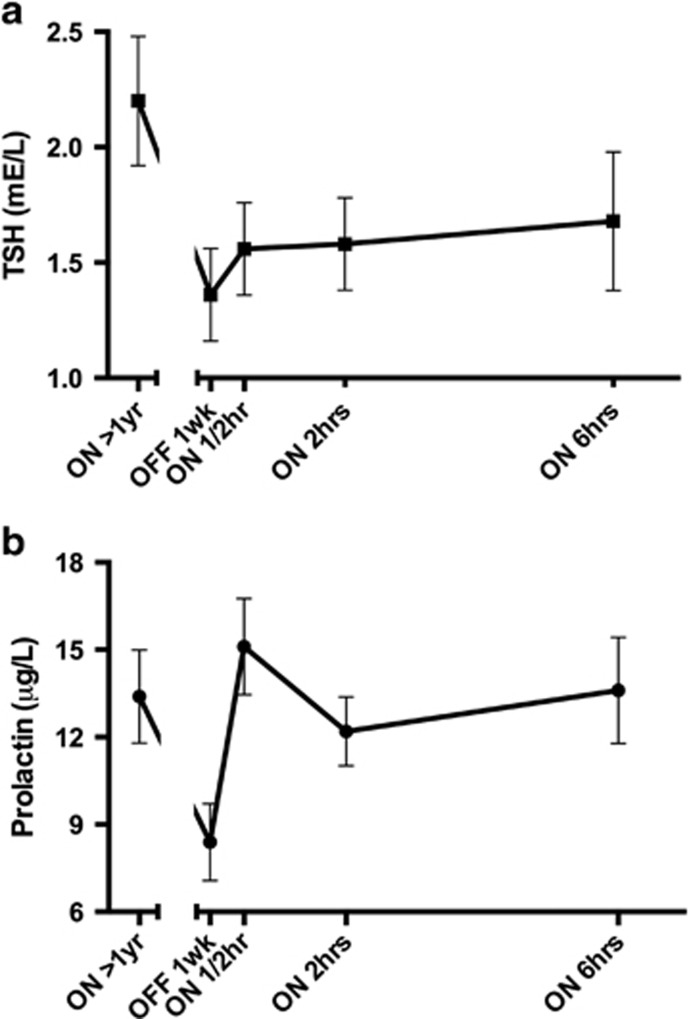
(**a**) Time course of deep brain stimulation (DBS)-induced thyroid-stimulating hormone (TSH) changes (mean±s.e.m.). (**b**) Time course of DBS-induced prolactin changes (mean±s.e.m.).

The mean TSH plasma levels decreased by 61% (*P*=0.003) from DBS ON to DBS OFF (Table 1 and Figure 3a), whereas the mean prolactin levels decreased by 59% (*P*=0.003; Figure 3b) and the median GH levels were 360% lower during OFF relative to active stimulation, although the difference failed to reach statistical significance.

We found no significant changes in the mean copeptin levels.

Finally, the mean HVA levels decreased by 11% (*P*=0.02) from active stimulation to DBS OFF.

#### DBS reactivation

At 30 min and 2 h after DBS reactivation, the mean TSH levels increased (near significantly) by 15% (*P*=0.06) and 16% (*P*=0.06), respectively. Six hours after DBS reactivation TSH levels remained 23% higher (*P*=0.07) compared with DBS OFF (Figure 3a). DBS reactivation increased TSH to levels still within the reference range (0.5–5.0 mU l^−1^).

At 30 min, 2  and 6 h after DBS reactivation the mean prolactin levels increased significantly compared with DBS OFF by 79% (*P*=0.001), 45% (*P*=0.000) and 62% (*P*=0.001), respectively (Figure 3b). According to a subgroup analysis, the results for TSH (*P*>0.5) and prolactin (*P*>0.1) were independent of sex.

We found no significant changes after DBS reactivation for GH or copeptin. Six hours after DBS reactivation HVA levels had increased to 31% (*P*=0.09), although the difference failed to reach a statistical significance.

#### Correlations

Y-BOCS, HAM-A, HAM-D or VAS symptom scores were not correlated with any of the endocrine changes during DBS ON to DBS OFF or after DBS reactivation. Neither did we find any significant correlation between changes of clinical symptoms and endocrine changes.

A subgroup analysis yielded no significant difference in DBS-induced neuroendocrine changes between DBS responders and non-responders.

We found a strong negative association between prolactin and HVA levels during long-term DBS ON (rs=−0.667, *P*=0.007). Furthermore, the increase in plasma TSH and prolactin, 30 min after DBS reactivation, was strongly correlated (*R*=0.682, *P*=0.005).

## Discussion

We believe we are the first to investigate the rapid effects of DBS reactivation. Our results demonstrate that after 1 week of DBS discontinuation, DBS reactivation results in a rapid and simultaneous ±50% improvement of anxiety, depression and obsessive-compulsive symptoms in 8 out of 10 initial DBS responders. Furthermore, we found that active DBS is associated with a rapid increase in neuroendocrine hormones compared with DBS OFF, although we found no significant correlation between clinical symptoms and neuroendocrine outcomes.

Interestingly, three out of five participants who failed to show a clinically relevant response after the first year of DBS still showed 50% symptom decrease within the first 2 h of DBS reactivation. These findings appear to be in line with several clinical observations reporting acute, but transient DBS effects in eventual non-responders during the DBS optimization phase or after DBS reactivation following a period of DBS cessation. One participant from the current study even turned off the stimulator every evening, reactivating it every next morning, in order to induce a transient improvement of affective symptoms that subsequently lessened his OCD symptoms during daytime. These findings suggest that DBS is capable of inducing rapid psychiatric symptom changes through an alternative or additional underlying mechanism.

In parallel with the acute clinical effects of DBS reactivation, discontinuation of DBS results in neuroendocrine changes that reversed acutely after DBS reactivation. TSH and prolactin decreased after switching off the DBS and increased again, although TSH increased to a lesser extent, within 30 min after DBS reactivation. This suggests that DBS changes are mediated, at least in part, via the hypothalamus, as both TSH and prolactin secretion are stimulated by hypothalamic thyrotropin-releasing hormone (TRH) and inhibited by dopamine released from neuroendocrine neurons projecting to the median eminence of the hypothalamus.^9, 10^ The strong negative association between HVA and prolactin levels in our study suggests the involvement of dopamine in the DBS changes, which is in agreement with our previous demonstration of DBS-induced dopamine release.^11^ However, a stimulatory effect of TRH on TSH and prolactin by DBS cannot be excluded at present especially as we found no association of TSH with HVA levels.

Interestingly, a significant body of preclinical and human data indicates that intravenous or intrathecal TRH administration has antidepressant effects, with some studies even reporting a mean HAM-D decrease of >50%.^12, 13^ Although responses to TRH administration were rapid and clinically robust, they only lasted for several days. In the study by Marangell *et al.*^12^ mood elevation coincided with a rapid increase in both plasma prolactin and TSH after intrathecal TRH administration. TRH-induced transient mood elevation is especially interesting as stimulation initiation in DBS for OCD is associated with mood elevation and hypomanic symptoms, also lasting for several days (Luigjes J *et al.* Hypomania as side effect of DBS in psychiatric patients, submitted).^2^ Therefore, we hypothesize on the basis of the present findings that TRH secretion may be a major determinant of the transient mood changes during DBS for the treatment of OCD.

A limitation of our study was the small samples size, which is, however, common for DBS studies. Furthermore, our design lacks a healthy control group; however, each subject served as his/her own control. As participants were aware of their stimulation settings, we cannot exclude the pronounced effects of DBS reactivation being (partially) based on the sense of expectancy or the recognition of symptom improvement. The small sample size did not permit us to exclude several confounding factors, including medication use or comorbid psychiatric disorders. Although three participants used clomipramine during DBS ON and OFF conditions, clomipramine treatment is associated with a significant decrease in TSH concentrations in OCD patients.^14^

Despite these limitations, the findings of the present study may stimulate further research on neuroendocrine changes of DBS, especially with a focus on TRH as a potential initiator in the mechanism of rapid symptom improvement and transient mood elevation.

## Figures and Tables

**Table 1:** Overview of DBS-induced neuroendocrine changes

	*Sample 1 DBS ON (>1 year)*	*Sample 2 DBS OFF (1 week)*	P*-value 1→2*	*Sample 3 DBS ON (1/2 h)*	P*-value 2→3*	*Sample 4 DBS ON (2 h)*	P*-value 2→4*	*Sample 5 DBS ON (6 h)*	P*-value 2→5*
TSH (mE l^−1^)	2.20 (±1.10)	1.36 (±0.78)	0.003**	1.56 (0.80)	0.057	1.58 (0.98)	0.037*	1.68 (1.21)	0.070
Prolactin (μg l^−1^)	13.40 (6.22)	8.43 (5.11)	0.003**	15.13 (6.42)	0.001**	12.25 (4.44)	0.000**	13.65 (6.59)	0.001**
GH (mE l^−1^)	3.6 (0.1–24.5)	1.0 (0.1–30)	0.112	2.7 (0.3–29)	0.629	1.1 0.2–12.6	0.706	0.8 (0.1–7.2)	0.239
Copeptin (pmol l^−1^)	4.9 (1.9–79.3)	6.2 (1.4–28.6)	0.158	5.0 (0.6–30.5)	0.451	5.3 (1.3–28.6)	0.798	5.2 (1.7–29.2)	0.410
HVA (nmol l^−1^)	10.42 (3.67)	9.33 (3.38)	0.018*	9.63 (3.02)	0.722	10.06 (6.93)	0.727	12.30 (5.29)	0.097

Abbreviations: DBS, deep brain stimulation; GH, growth hormone; HVA, homovanillic acid; max, maximum; min, minimum; TSH, thyroid-stimulating hormone.

Values are the mean levels (s.d.). For GH and copeptin we present the median levels (min–max). *P≤0.05; **P<0.01.
